# Long AKAP18 isoforms anchor ubiquitin specific proteinases and coordinate calcium reuptake at the sarcoplasmic reticulum

**DOI:** 10.1016/j.jbc.2025.110317

**Published:** 2025-05-29

**Authors:** Taeyeop Park, Katherine Forbush, Yong Li, Oscar Vivas, Kacey J. Rosenthal, Jerome Falcone, Cassandra J. Wong, James E. Bruce, Claudia Moreno, Carmen W. Dessauer, John D. Scott

**Affiliations:** 1 Department of Integrative Biology and Pharmacology, McGovern Medical School at University of Texas Health Science Center Houston, Houston, Texas, USA; 2 Department of Pharmacology, University of Washington School of Medicine, Seattle, Washington, USA; 3 Lunenfeld-Tanenbaum Research Institute, Sinai Health, Toronto, Ontario, USA; 4 Department of Genome Sciences, University of Washington School of Medicine, Seattle, Washington, USA; 5 Howard Hughes Medical Institute, Department of Neurobiology and Biophysics, University of Washington School of Medicine, Seattle, Washington, USA

**Keywords:** A-kinase anchoring protein, cardiomyocyte, deubiquitinase, protein kinase A, signal transduction

## Abstract

Subcellular targeting of signaling enzymes influences where and when various modes of intracellular communication operate. Macromolecular complexes of signal transduction and signal termination elements favor reversible control of repetitive processes. This includes adrenergic stimulation of excitation–contraction coupling in the heart. Long isoforms of A-kinase anchoring protein 18 (AKAP18**γ** and **δ**) modulate this process *via* regulation of calcium uptake into the sarcoplasmic reticulum through the Ca^2+^ATPase 2a (SERCA2a). AKAP18 proximity-proteomic screening in cardiomyocytes identifies networks for protein kinase A (PKA) and ubiquitin-specific proteinases (USPs). A 2′phosphoesterase domain on AKAP18 interfaces with the USP4 isoform at the Z bands of sarcomeres. PKA stimulates USP4 activity in the presence of the anchoring protein. AKAP18 anchored PKA phosphorylates serine 829 on USP4, a conserved residue near the active site of this deubiquitinase. Antibodies against the pSer^829^ motif show that adrenergic stimulation enhances phosphorylation of USP4 in mouse adult cardiomyocytes. In related studies, elevated USP4 phosphorylation at Ser^829^ is detected in human post myocardial infraction tissue as compared to healthy tissue. Thus, phosphorylation of sarcoplasmic USP4 may be a cardioprotective response. Pharmacological inhibition of PKA or deletion of the *AKAP7/18* gene in mice decreases calcium flux through the exchanger. This suggests that loss of the anchoring protein impacts SERCA2 action. Thus, AKAP18/PKA/USP4 complexes are well positioned to influence the rate and magnitude of calcium reuptake during the cardiac cycle.

The effect of intracellular signals often proceeds through the posttranslational modification of proteins (1). Protein kinases catalyze phosphotransferase reactions that elicit transcriptional activation, protein synthesis, and cell division (2, 3, 4). These enzymes partner with phosphoprotein phosphatases that remove phosphate from substrates (5, 6). Protein ubiquitination, the attachment of the 8.6 kDa ubiquitin modifier to target proteins, is another frequently used covalent modification. This signaling machinery drives protein processing, immune responses, and programmed cell death (7). Ubiquitination utilizes three classes of enzymes. These are ubiquitin-activating enzyme (E1), ubiquitin conjugating enzymes (E2), and ubiquitin ligases (E3). To counteract this process, deubiquitinating enzymes (DUBs) cleave ubiquitin from target proteins and disassemble polyubiquitin chains (8). Less is known about the subcellular organization of DUB enzymes (9).

Organellar targeting of signaling enzymes proceeds through association with anchoring proteins (10, 11). A-kinase anchoring proteins (AKAPs) are well-studied scaffolding elements that sequester protein kinase A (PKA) and other signaling enzymes at defined subcellular locations (12, 13, 14). Over 70 AKAPs have been identified in the human proteome (15, 16, 17). AKAPs contain a common amphipathic helix that docks into a reciprocal binding furrow on the surface of the R subunit dimer within the PKA holoenzyme (18, 19). Structural studies have revealed the modular nature of AKAP-PKA macromolecular assemblies (19, 20, 21, 22). Accordingly, PKA signaling emanates from “AKAP signaling islands” with sharply defined radii of action (17, 23, 24). New features of this revised model include 1) compartmentalized cAMP synthesis; 2) retention of cAMP within insulated nanodomains, and 3) preservation of kinase activity within the AKAP-PKA holoenzyme complex (25, 26, 27, 28). The physiological significance of this mechanism is validated in numerous contexts (15, 29, 30, 31, 32). Furthermore, perturbed local PKA signaling and lesions in AKAP genes are linked to a growing number of diseases (33, 34, 35, 36, 37).

Adrenergic stimulation and mobilization of cAMP supports excitation–contraction coupling in the heart (38). Each phase of this vital process is influenced by different anchoring proteins. AKAP79/150 and AKAP18⍺ and β isoforms (products of the *AKAP5* and *AKAP7* genes, respectively) direct phosphorylation of the L-type Ca^2+^-channel and its ancillary regulatory proteins (39, 40). The muscle selective anchoring protein mAKAP synchronizes bidirectional control of RyR/calcium release channels on the sarcoplasmic reticulum (41). Cardiac troponin I (cTnI) influences PKA phosphorylation of myofilament proteins (42). Removal of calcium during myocyte relaxation involves the sarcolemmal Ca^2+^ ATPase (sarcoplasmic reticulum through the Ca^2+^ATPase, SERCA). Long isoforms of AKAP18 (**γ** and δ) favor PKA phosphorylation of phospholamban and SERCA2a to enhance Ca^2+^ reuptake (43) AKAP18 associated phosphodiesterase 3A terminates this later phase of excitation–contraction coupling (44, 45, 46, 47, 48, 49). Repolarization of cardiac action potentials (APs) involves the anchoring protein Yotiao that facilitates modulation of IKs potassium channels that go awry in long-QT syndrome arrythmias (37, 50, 51).

Heart failure has been attributed to changes in the phosphorylation status and proteostasis of SERCA2 (52, 53, 54). Glycogen synthase kinase 3 phosphorylation of Ser 663 on SERCA2 occurs during myocardial infarction (55, 56). Interestingly conjugation with the small ubiquitin-like modifier 1 (SUMO-1) stabilizes the protein, whereas ubiquitination of SERCA2 is a pathological outcome that leads to proteasomal degradation (57). This latter event is reversed by deubiquitinases (58, 59). Herein, we report that AKAP18 γ and δ form a sarcoplasmic anchor for ubiquitin specific proteinases including the ubiquitin-specific proteinase 4 (USP4) isoform. Clustering with PKA permits phosphorylation of serine 829 on USP4 to enhance deubiquitinase activity. Elevated levels of phospho-USP4 in post myocardial infraction tissue may represent a cardioprotective event to reduce ubiquitin on sarcomere proteins.

## Results

### AKAPs associate with DUB enzymes

The ubiquitin-proteasome system impacts the action of cardiac cells. DUB enzymes fulfill a signal termination capacity by liberating ubiquitin from the surface of target proteins (8). Ubiquitination of SERCA2 by enzymes such as UBR3 or UBR6 facilitates depletion of this calcium ATPase, whereas deubiquitination is a cardioprotective event that favors protein stability (58, 59, 60). Hence, we reasoned that scaffolding proteins such as AKAPs may sequester deubiquitinases in proximity of SERCA2.

**Figure 1:**
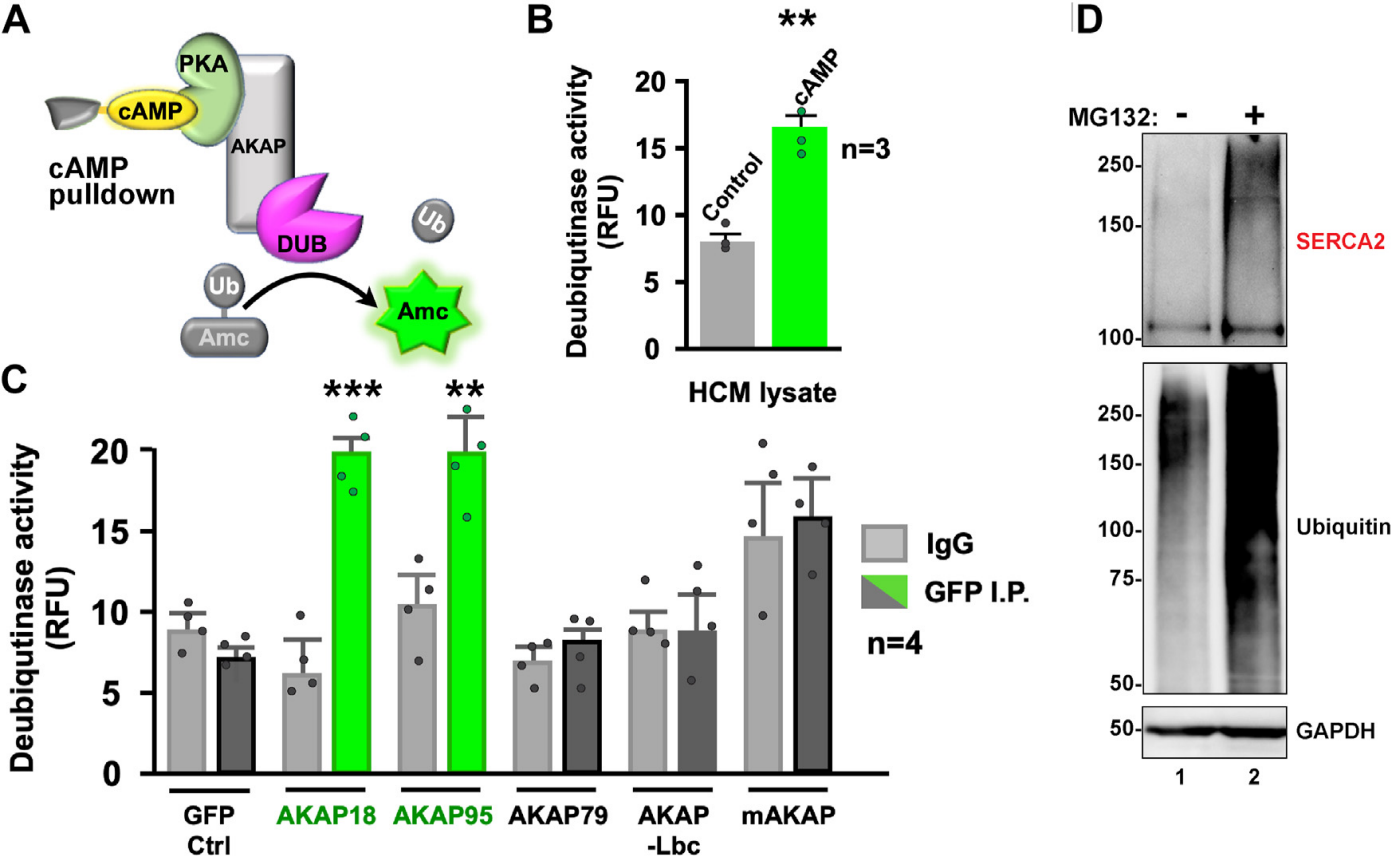
**AKAP18 interfaces with the ubiquitin machinery.***A*, schematic of screen for AKAP associated deubiquitinases. R subunit-AKAP complexes were captured on cAMP-agarose. Deubiquitinase activity was measured by AMC-ubiquitin fluorescence assay. *B*, cofractionation of deubiquitinase activity (RFU) from control (*gray*) and cAMP (*green*) eluates. Data from three experiments are presented. *C*, cofractionation of endogenous deubiquitinase activity with AKAP-GFP fusions (named below columns) in HEK293 cells. Activity measurements by AMC-ubiquitin fluorescence assay (RFU). IgG (*gray*) and GFP (*charcoal*) immune complexes are indicated. Deubiquitinase activities above background (*green*) are indicated. Data from four experiments are presented. *D*, ubiquitinated proteins were captured on TUBE2-agarose from cardiomyocyte lysates pretreated with DMSO vehicle (lane 1) or MG132 (lane 2). Both fractions were eluted from the affinity resin by boiling in SDS and separated by PAGE gels. Immunoblot detection shows (*top*) SERCA2a; high molecular weight bands consistent with polyubiquitination are evident (*mid*) total ubiquitin, (*bottom*) GAPDH loading control. Molecular weight markers are indicated. *t* Test and one-way ANOVA were used to test for statistical significance. *p*-values <0.05 were deemed statistically significant. AKAP18, A-kinase anchoring protein 18; AMC, 7-amido-4-methylcoumarin; DMSO, dimethyl sulfoxide; RFU, relative fluorescence unit; SERCA2a, sarcoplasmic reticulum through the Ca^2+^ATPase 2a.

We initiated an enzymatic screen for AKAP-associated deubiquitinases in human cardiomyocyte (HCM) extracts. AKAPs were fractionated by virtue of the ability of their R subunit binding partners to tightly interact with cAMP-agarose (Fig. 1*A*). Immobilized protein was eluted with 500 μM cAMP, and deubiquitinase activity was measured using a commercially available ubiquitin-7-amino-4-methylcoumarin (Ub-AMC) fluorescent assay (61). Deubiquitinase activity was enriched 2.1 ± 0.2-fold (n = 3) over control lysates eluted with 5′ AMP (Fig. 1*B*, green). This argues that deubiquitinases are present within AKAP complexes. Experiments were repeated in another cell line to ascertain which AKAPs retained deubiquitinase activity. HEK293 cells were transfected with GFP and similarly tagged constructs encoding the cardiac anchoring proteins AKAP18, AKAP95, AKAP79, AKAP-Lbc, or mAKAP (62, 63, 64, 65). AKAP-GFP complexes were isolated by immunoprecipitation and deubiquitinase activity was measured by Ub-AMC fluorescent assay (Fig. 1*C*). Deubiquitinase activity was enriched over immunoglobulin G (IgG) controls only in AKAP18 and AKAP95 immune complexes (Fig. 1*C*, green columns; n = 4). These results corroborated independent proteomic screens showing AKAP18 and AKAP95 interact with the ubiquitin specific proteinases USP4 and USP49, respectively (66).

Two factors focused our analyses toward AKAP18 interaction with deubiquitinases. First, this anchoring protein coordinates PKA phosphorylation of SERCA2 and its modulator protein phospholamban (43). Second, enrichment of ubiquitinated proteins from HCM lysates by affinity chromatography on (**t**andem **u**biquitin **b**inding **e**ntities) TUBE2-agarose revealed ubiquitination of SERCA2 (Fig. 1*D*). Immunoblot detection of SERCA2 revealed high molecular weight bands that are consistent with polyubiquitination (Fig. 1*D*, top panel). This is particularly evident upon treatment with the proteasomal inhibitor MG132 (Fig. 1*D*, top panel; lane 2). As expected, immunoblot detection of ubiquitinated proteins was enriched in the presence of MG132 (Fig. 1*D*, mid panel; lane 2). GAPDH served as a loading control (Fig. 1*D*, bottom panel). Collectively, data in Figure 1 indicate that SERCA2 is ubiquitinated and imply that AKAP18 associated deubiquitinases may serve to remove this covalent modification from the surface of the calcium exchanger.

**Figure 2:**
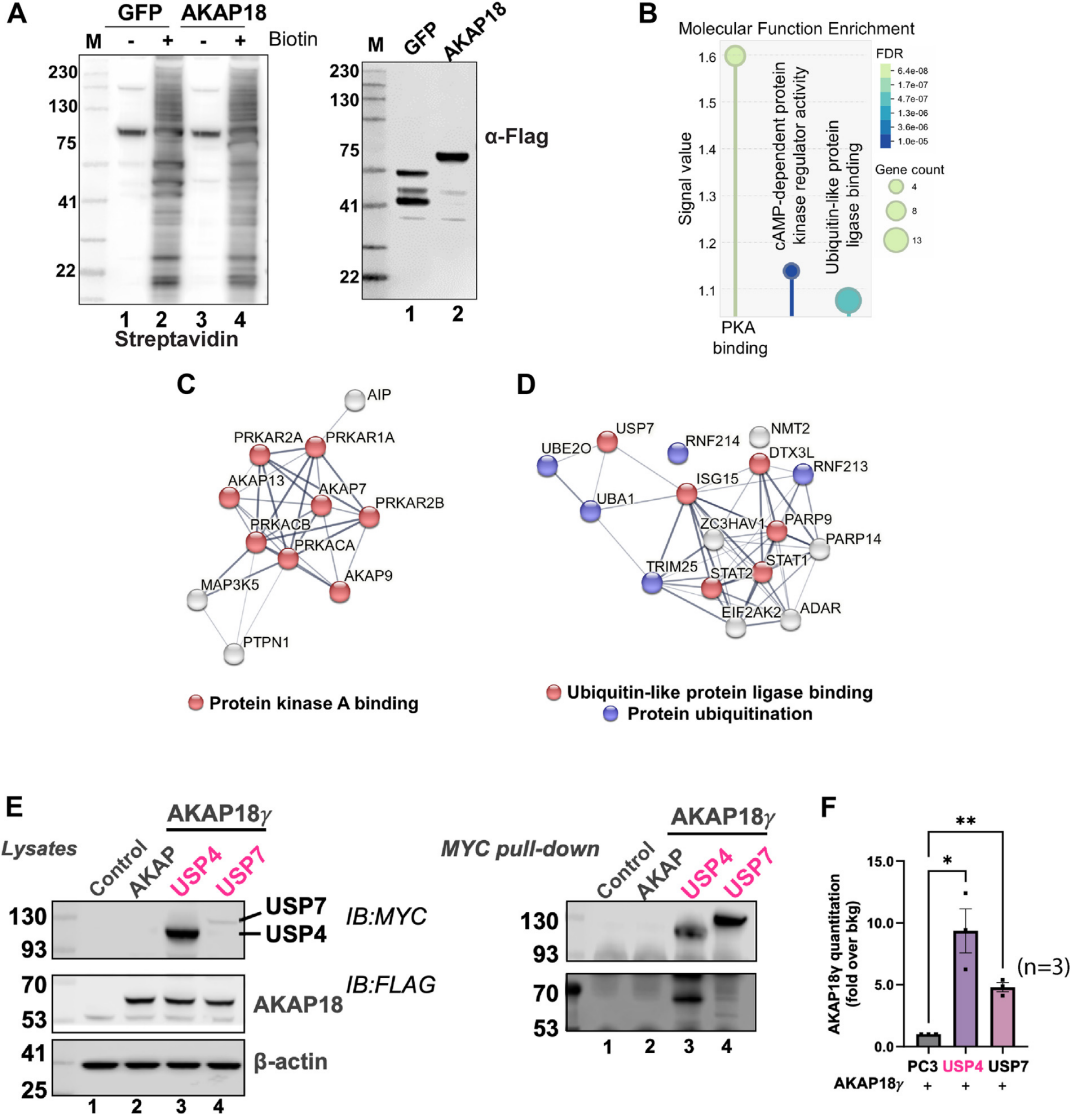
**AKAP18 recruits ubiquitin specific proteinases.***A*, validation and proteomic identification of AKAP18 interactors in neonatal cardiomyocytes. Immunoblot shows the expression and biotinylation patterns of GFP-miniTurbo (lanes 1 and 2) and AKAP18-miniTurbo (lanes 3 and 4). Cells were treated ± biotin. Biotinylated proteins were detected using streptavidin-HRP. Representative image is shown from three independent experiments. Molecular weight markers are indicated. *B*, molecular function gene ontology groups for AKAP18 (SS ≥ 0.7). *C*, STRING analysis (Ver 12.0) associated with PKA binding (GO:0051018) and (*D*) ubiquitin-like protein ligase binding (GO:0044389) clusters. *E*, plasmids encoding FLAG-tagged AKAP18γ-mini turbo was coexpressed with empty vector (pCDNA3) or plasmids encoding MYC-tagged USP4 or MYC-tagged USP7 in HEK293 cells. Expression of USP (lanes 3 and 4) and AKAP18γ (lanes 2, 3, and 4) was confirmed by immunoblot analysis of whole-cell lysates, with β-actin serving as a loading control (*left panel*). Complex formation was assessed by pull-down of USPs using anti-MYC antibody and detection by Western blot of FLAG-tagged AKAP18γ (lanes 3 and 4, *middle panel*). *F*, quantification of AKAP18γ complex formation with USPs from three independent experiments by densitometric analyses is presented in the graph form. Data shown as the mean ± SEM from three biological replicates. Statistics: paired *t* test using nonnormalized values, ∗∗*p* < 0.01, ∗*p* < 0.05. AKAP18, A-kinase anchoring protein 18; HRP, horseradish peroxidase; PKA, protein kinase A; USP, ubiquitin-specific proteinase.

To generate near-neighbor protein interaction maps of AKAP18, we performed proximity-proteomics in primary rat neonatal cardiomyocytes. Cells were engineered to express the miniTurbo biotin ligase fused to the C terminus of AKAP18γ (16). MiniTurbo fused to GFP served as a control. Immunoblot detection of biotinylated proteins in cell lysates revealed distinct labeling patterns in cells expressing either construct (Fig. 2*A*, left panel; lanes 2 and 4). Immunoblot detection of miniTurbo fusion proteins served as loading controls (Fig. 2*A*, right panel; lanes 1 and 2). Biotinylated proteins were captured on streptavidin beads and identified by mass spectrometry (MS). Data were analyzed by significance analysis of interactome (SAINT; score cutoff of ≥0.7 (BFDR≤0.03)). This algorithm calculates the probability that high-confidence protein interactors are enriched above GFP control samples. Proximity MS identified 115 near neighbors of AKAP18 (Fig. S1). Search tools for recurring instances of neighboring genes (STRING analysis) identified the top three molecular function gene ontology groups as PKA binding (GO:0051018), cAMP-dependent protein kinase regulator activity (GO:0004691), and ubiquitin-like protein ligase binding (GO:0044389, Figs. 2, *B*–*D*, and S1). This screen identified several elements of the protein ubiquitin machinery including ubiquitin ligases UBE2O, UBA1, and RNF213, and the DUB enzyme USP7 (Fig. 2*D*). Independent validation of AKAP18 interactions with USP4 and USP7 were carried out in HEK293 cells (Fig. 2*E*). Cells were cotransfected with MYC tagged vectors encoding USP4 or USP7 and FLAG-tagged AKAP18 (Fig. 2*E*, left panels, lanes 2–4). Immunoblot analyses of MYC-pulldowns confirmed cofractionation of the anchoring protein with each deubiquitinase (Fig. 2*E*, right panels, lanes 2–4). Quantitation of three independent experiments confirmed that the AKAP18/USP4 protein-protein interaction was more robust than USP7 association with the anchoring protein (Fig. 2*F*). For this reason, and evidence from our other proteomic screens, we chose to focus on investigating AKAP18/USP4 interactions in the regulation of cardiac signaling (17). Furthermore, USP7 is a specialized enzyme more often utilized for mitochondrial biogenesis and myogenin stability in skeletal muscle (67, 68, 69).

### SERCA2 associates with USP4

**Figure 3:**
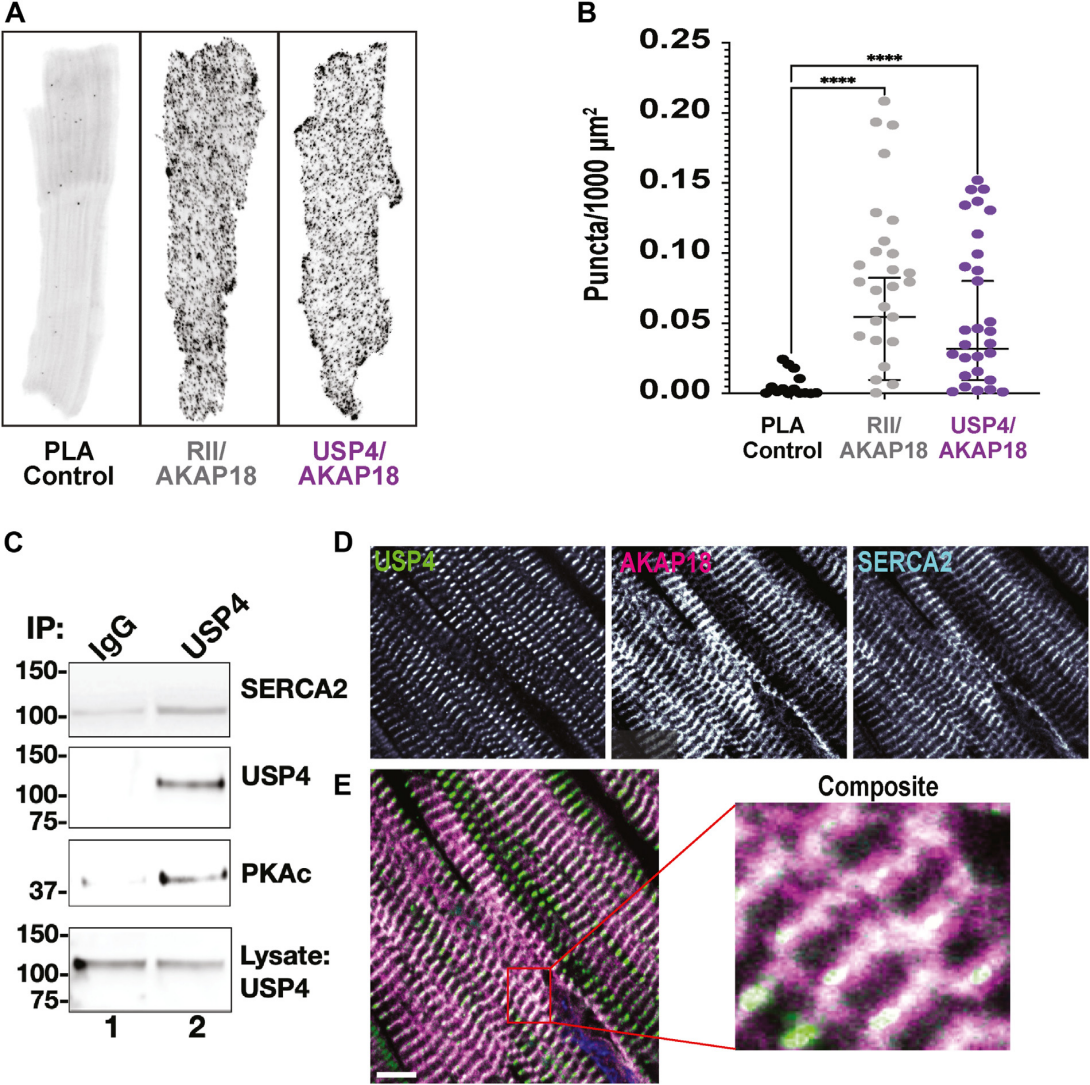
**Validation of AKAP18 interaction with USP4.***A*, proximity ligation assay (PLA) shows (*left*) control, (*mid*) RII-AKAP18 and (*right*) USP4-AKAP18 puncta in representative mouse adult cardiomyocytes. *B*, quantification of PLA puncta per 1000 μm^2^ from five cells of each condition using Fiji/ImageJ. *C*, IgG control (lane 1) and USP4 (lane 2) immune complexes isolated from HMC cells. Immunoblot detection of (*top*) SERCA2, (*upper-mid*) USP4 and (*lower mid*) PKAc. Immunoblot detection of USP4 (*bottom*) serves as a loading control. Molecular weight markers are indicated. *D*, immunofluorescence detection of USP4 (*green*), AKAP18 (*magenta*) and SERCA2 (*cyan*) in paraformaldehyde fixed section of human heart tissue. *E*, composite staining pattern. The scale bar represents 20 μm. (Inset) composite imaging showing codistribution of three signals (*white*) at higher magnification. All measurements presented as means ± SEM. AKAP18, A-kinase anchoring protein 18; IgG, immunoglobulin G; PKAc, protein kinase A catalytic subunit; PLA, proximity ligation assay; SERCA2, sarcoplasmic reticulum through the Ca^2+^ATPase 2a; USP, ubiquitin-specific proteinase.

Three cell-based approaches validated the AKAP18/USP4 interaction. First, proximity ligation assays (PLAs) detect endogenous protein-protein interactions that occur within 40 to 60 nm (25). Isolated mouse adult cardiomyocytes were fixed and stained with antibodies against the RII subunits of PKA, AKAP18, and USP4. Samples were subjected to the PLA amplification protocol before imaging of PLA puncta (Fig. 3, *A* and *B*). Few PLA puncta were detected in control cardiomyocytes treated with secondary antibodies alone (Fig. 3*A*, left panel & 3B). In contrast, robust signals were detected when PLA experiments were performed with RII and AKAP18 antibody pairs (gray) and USP4 and AKAP18 antibody pairs (purple, Fig. 3*A*). Quantification of (puncta/100 μm^2^) from discrete regions on five individual cells from each experimental group are presented in Figure 3*B*. Second, fractionation of USP4 with SERCA2 was confirmed by coimmunoprecipitation. USP4 immune complexes were enriched with SERCA2 and the PKA catalytic subunit (PKAc) as compared to IgG controls (Fig. 3*C*, top and lower mid panels, lane 2). Additional controls confirmed enhanced detection of USP4 in immune complexes (Fig. 3*C*, upper mid panel, lane 2). Immunoblot detection of the deubiquitinase in cell lysates serves as a loading control (Fig. 3*C*, lower panel). Third, immunofluorescent analyses of normal human heart tissue sections detected distinct but overlapping USP4 (green), AKAP18 (magenta), and SERCA2 (cyan) signals that were concentrated at the Z bands of cardiomyocytes (Fig. 3*D*, upper panels). Codistribution of these signals was evident at higher magnifications (Fig. 3*E* lower panel and composite inset). Data in Figure 3 show that AKAP18 sequesters PKA and USP4 at lateral boundaries of the sarcomere.

### Defining the AKAP18/USP4 interface

**Figure 4:**
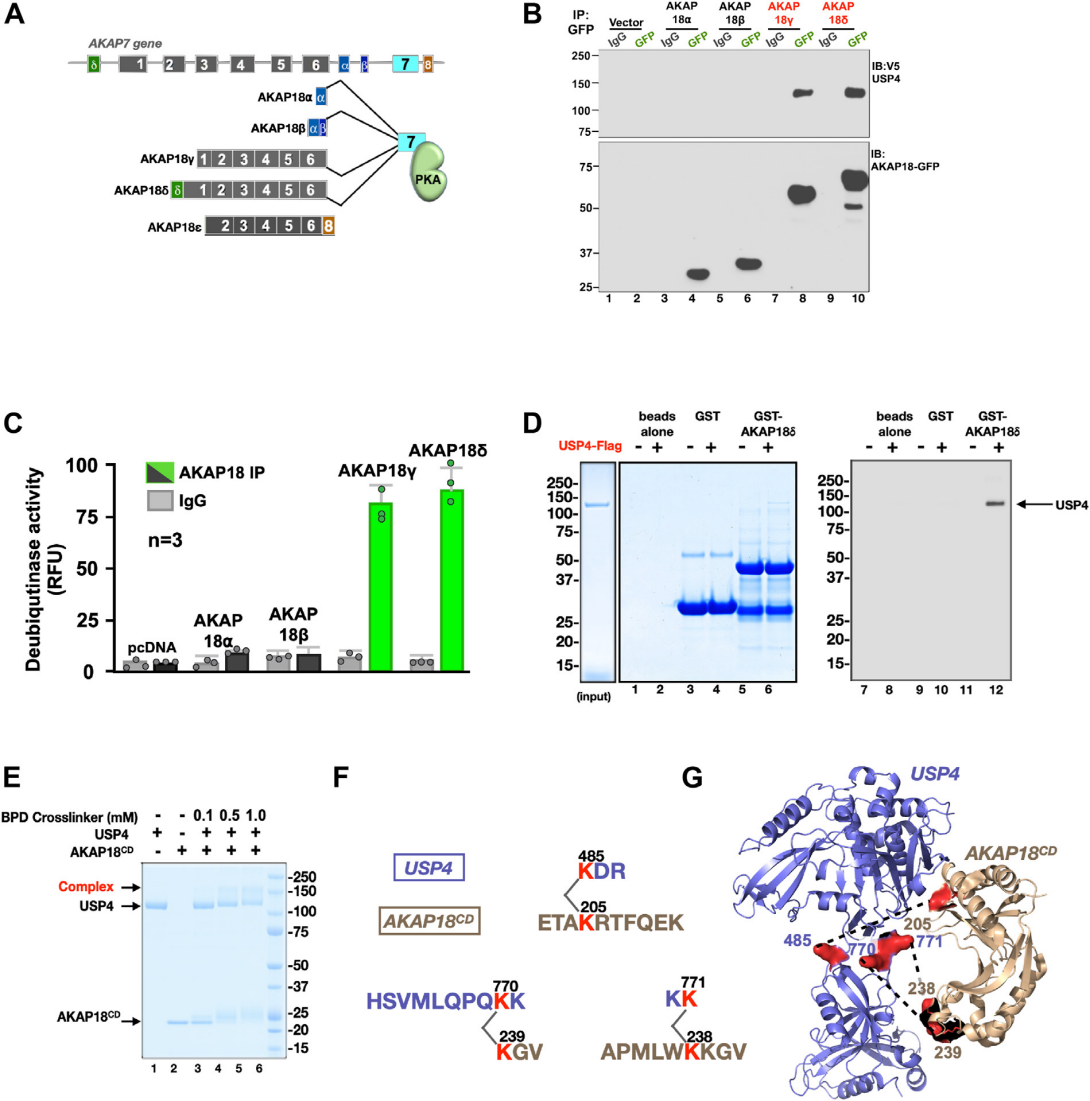
**The central domain of AKAP18 interfaces with USP4.***A*, schematic of AKAP7 gene organization depicting exon–intron structure and alternatively spliced isoforms. Exon 7 (*cyan*) encodes the PKA anchoring domain. *B* and *C*, AKAP18 γ and δ isoforms bind USP4. GFP tagged AKAP18 isoforms were expressed with V5 tagged USP4 in HEK293 cells. *B*, immunoblot detection of (*top panel*) V5-USP4 and (*lower panel*) AKAP18 isoforms in GFP immune complexes. Immunoblot detection of IgG controls (lanes 1, 3, 5, 7, and 9) are included. Molecular weight markers are indicated. *C*, activity measurements of immune complexes by AMC assay (RFU). IgG fractions (*gray*) and GFP (*charcoal*) are indicated. Deubiquitinase activities above background (*green*) are specified. Data from three experiments is presented. *C*, coomassie blue staining of (*left*) purified Flag-USP4 and (*mid*) GST and GST-AKAP18δ proteins. AKAP18δ pull-down of USP4 is visible (*mid panel*, lane 8). (*Right panel*) AKAP18δ pull-down of USP4 confirmed by immunoblot detection (lane 12). *E* and *F*, chemical cross-linking studies and molecular modeling of the USP4/AKAP18 interface. *E*, chemical cross-linker BDP (concentrations indicated above each lane) incubated with purified USP4 with the central (CD) domain of AKAP18. Coomassie blue stained gel revealing cross linked protein complexes. USP4, AKAP18^CD^ and protein complex are indicated (*F*) Amino acid sequence (one letter code) of cross linked peptide for USP4 (*purple*) and AKAP18^CD^ (*gold*). The position of modified lysine’s (*red*) is denoted. Molecular weight markers are indicated on all gels and blots. *G*, molecular model using PDB coordinates for USP4 (3JYU, *purple*) and AKAP18^CD^ domain (2VFK, *gold*) to simulate the docking of both proteins. Positions of cross-linked lysines (*red*) are indicated. AKAP18, A-kinase anchoring protein 18; AMC, 7-amido-4-methylcoumarin; PDB, protein data bank; PKA, protein kinase A; RFU, relative fluorescence unit; USP, ubiquitin-specific proteinase.

AKAP18 is a family of five alternatively spliced isoforms encoded by the *AKAP7* gene (Fig. 4*A*; (16, 62, 70)). Short isoforms (AKAP18⍺ and β) encode lipid modified anchoring proteins that participate in cAMP dependent modulation of ion channels (62, 71). Long isoforms (AKAP18γ and δ) contain a 2′phosphoesterase domain and target PKA to various intracellular structures including sarcomeres (67, 72). Nuclear AKAP18ε lacks a PKA-anchoring helix ((16); Fig 4*A*). Characterization of the AKAP18-USP4 interface was conducted in several ways. HEK293 cells were cotransfected with vectors encoding GFP-AKAP18 isoforms and V5-tagged USP4. Immunoprecipitation of each AKAP18 isoform was confirmed by immunoblot (Fig. 4*B*, lower panel, lanes 4–10). Only the AKAP18 γ and δ isoforms interact with USP4 (Fig. 4*B*, upper panel, lanes 8 & 10). Enzymological validation used the Ub-AMC fluorescent assay (Fig. 4*C*). Deubiquitinase activity was enriched 7.5 ± 1.1 and 9.2 ± 1.3-fold over IgG controls in AKAP18γ and AKAP18δ immune complexes respectively (Fig. 4*C*, green columns; n = 3).

*In vitro* analyses utilized purified AKAP18δ and FLAG tagged-USP4 proteins (Fig. 4, *D*–*F*). Glutathione-*S*-transferase-pull-down experiments show that USP4 binds directly to AKAP18δ when protein mixtures are separated by SDS PAGE (Fig. 4*D*, center panel, lane 6). Immunoblot analyses with anti-FLAG antibodies confirmed the presence of the deubiquitinase (Fig. 4*D*, right panel, lane 12). These findings argue that AKAP18 isoforms containing the central 2′phosphoesterase domain (CD) interact with USP4 (72, 73). Chemical cross-linking studies and molecular modeling support this conclusion. A protein interaction reporter (PIR) cross-linker known as BDP has reactivity toward ε amino group of lysines allowing its use as a heterobifunctional cross-linking agent (74, 75). DSS was used over a range of concentrations (0.1–1.0 mM) to probe the USP4/AKAP18^CD^ interface (Fig. 4*E*, lanes 4–6). Trypsinized peptides were separated by liquid chromatography, and MS detected cross-linked lysine residues using the xQuest algorithm (76). Three peptides heterodimeric cross-linked peptides (K485 of USP4 to K205 of AKAP18; K770-K239 and K771-K238) were mapped (modified lysines in red; Fig. 4*F*). These results were consolidated into a molecular model of the USP4-AKAP18^CD^ interface using protein data bank (PDB) coordinates from crystal structures of both proteins (Fig. 4*G*). Contact sites on both flanks of the 2′phosphoesterase domain (gold) interface with a disordered linker region between the catalytic core and *DUSP* (domain present in ubiquitin-specific protease) motif of USP4 (purple, Fig. 4*G*).

### USP4 is phosphorylated by AKAP18 anchored PKA

**Figure 5:**
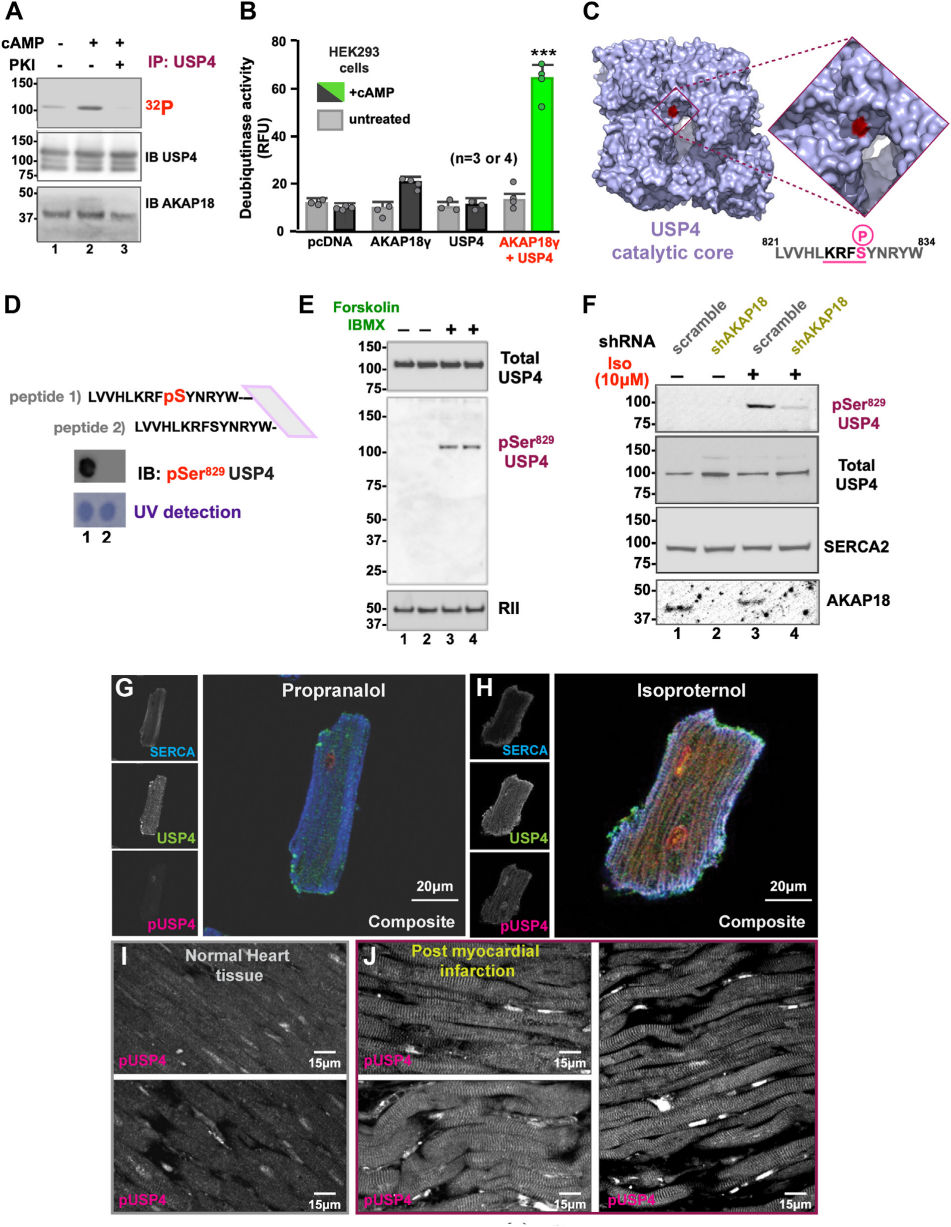
**USP4 is phosphorylated by AKAP18 anchored PKA.***A*, autoradiograph detecting (*top*) ^32^P phosphate incorporation into USP4 immune complexes. Immunoblots confirmed the presence of USP4 (*mid*) and AKAP18 (*bottom*). Experiments were performed without agonist (lane 1) or in the presence of cAMP (lanes 2 and 3), and the peptide kinase inhibitor (PKI). *B*, GFP tagged AKAP18γ and V5 tagged USP4 were expressed individually or together (indicated below each column) in HEK293 cells. Activity measurements by AMC assay (RFU) of untreated (*gray*) and 10 μm cAMP stimulated (*charcoal*) cells are presented. Deubiquitinase activity above background (*green*) is indicated. Data from four experiments are presented. *C*, space filling model of USP4 (3JYU, *purple*) showing the active site. Serine 829 (*red*) is indicated. (Inset; *top*) higher magnification of active site region. Serine 829 (*red*) is indicated. (Inset; *bottom*) Conserved consensus PKA motif between residues 821 to 834 of USP4. Serine 829 (*red*) is indicated. Amino acids indicated using one letter code (*D* and *E*) Characterization of phospho-USP4-Ser 829 antibodies. *D*, phospho (peptide 1) and nonphospho (peptide 2) analogs of the USP4 821 to 834 sequence were generated by Spot array synthesis. Affinity purified pSer^829^ antibodies were evaluated by immunoblot. UV detection of Trp 834 shows amounts of each immobilized peptide. *E*, maximal cAMP stimulation enhances detection of pSer^829^ on USP4. Immunoblot of (*top*) total USP4, (*mid*) pSer^829^ and (*bottom*) RII subunit of PKA. Analyses of lysates from unstimulated HCM (lanes 1 and 2) or cells treated with Forskolin/IBMX (10 μM, lanes 3 and 4) to maximize the cAMP response. *F*, shRNA mediated gene silencing of AKAP18γ and AKAP18δ was performed in HCM cells (lanes 2 and 4). Control cells were treated with scrambled RNA (lanes 1 and 3). Isoproterenol (10 μM) was used as a physiological agonist of the cAMP signaling. Immunoblot detection of (*top*) pSer^829^, (*upper-mid*) total USP4, (*lower mid*) SERCA2 and (*bottom*) AKAP18. Molecular weight markers are indicated for all autoradiographs and immunoblots. *G*–*J*, USP4 pSer^829^ antisera detects phosphorylation *in situ*. *G* and *H*, immunofluorescent detection of SERCA2 (*cyan*), total USP4 (*green*), and pSer^829^ (*magenta*) in paraformaldehyde fixed adult mouse adult cardiomyocytes. Cells treated with (*G*) beta-blocker drug propranolol and (*H*) β-agonist Isoproterenol. The scale bars (20 μm) are indicated. *I* and *J*, paraffin embedded tissue section of human heart was subjected to immunofluorescent analyses. Detection of pSer^829^ USP4 in (*I*) Normal heart and (*J*) seven day post myocardial infraction. Scale bars (15 μm) are indicated. AKAP18, A-kinase anchoring protein 18; AMC, 7-amido-4-methylcoumarin; HCM, human cardiomyocyte; PKA, protein kinase A; RFU, relative fluorescence unit; SERCA2, sarcoplasmic reticulum through the Ca^2+^ATPase 2a; USP, ubiquitin-specific proteinase.

We reasoned that incorporation within AKAP18-PKA signaling islands could favor phosphorylation of USP4. The deubiquitinase was immunoprecipitated from HCM cell lysates loaded with γ^32^P ATP. Immune complexes were monitored for ^32^P incorporation by autoradiography (Fig. 5*A*, top panel). Under basal conditions ^32^P incorporation into USP4 was modest (Fig. 5*A*, top panel, lane 1). Incorporation of ^32^P was augmented by cAMP stimulation (10 μM Forskolin/IBMX; Fig. 5*A*, top panel, lanes 2). This effect was blocked by the PKI 4 to 25 peptide, a selective inhibitor of PKA ((77); Fig 5*A*, top panel, lanes 3). Control immunoblots confirmed equivalent levels of USP4 and AKAP18 in each sample (Fig. 5*A*, mid and lower panels). This implies that localized PKA phosphorylates USP4. Orthogonal studies measured cAMP responsive changes in deubiquitinase activity (Fig. 5*B*). HEK293 cells were transfected with vectors encoding AKAP18γ, USP4, or a combination of both. Deubiquitinase activity was measured by Ub-AMC fluorescent assay (61). Upon cAMP stimulation, deubiquitinase activity was marginally elevated 2.3 ± 0.1-fold (n = 4) in the presence of the anchoring protein but remained unaltered upon introduction of USP4 alone (Fig. 5*B*). In contrast, deubiquitinase activity was augmented 5.4 ± 0.4-fold (n = 4) when USP4 was coexpressed with AKAP18γ (Fig. 5*B*, green). These results argue that PKA phosphorylation of USP4 stimulates deubiquitinase activity within the context of AKAP18 signaling islands.

Scansite identifies protein sequence motifs that are likely to be phosphorylated by specific protein kinases (78). Consensus PKA sites were identified at residues threonine 771, threonine 772, and serine 829 on USP4. Molecular modeling revealed that serine 829 resides in a cleft at the active site of USP4 (Fig. 5*C* and inset). This motif is conserved across species (Fig. 5*C*). Phosphopeptide antibodies were generated to characterize Ser 829 phosphorylation on USP4. Initial testing by peptide array monitored the specificity of affinity purified antibody. Anti-pSer^829^ detected only immobilized USP4 821 to 834 phosphopeptide at a 1:100 dilution (Fig. 5*D*, upper panel). UV detection of Trp 834 confirmed that equivalent amounts of phospho and non-phospho peptides were immobilized on the spot array (Fig. 5*D*, lower panel). Secondary validation was conducted using human adult cardiomyocytes. In unstimulated cells the pSer^829^ signal was not detected by immunoblot (Fig. 5*E*, mid panel, lanes 1 & 2). A strong pSer^829^ signal was evident in cells treated with 10 μM Forskolin/IBMX to maximize the cAMP response (Fig. 5*E*, mid panel, lanes 1 & 2). Control immunoblots confirmed equivalent amounts of total USP4 in each sample (Fig. 5*E*, top panel). Detection of RII subunits of PKA served as a loading control (Fig. 5*E*, bottom panel).

More stringent validation was conducted upon application of isoproterenol, a selective agonist of β-adrenergic receptors that increases heart rate and force of cardiac contraction (79). Under basal conditions the pSer^829^ signal was not detected by immunoblot (Fig. 5*F*, top panel, lanes 1 and 2). However, a robust pSer^829^ signal was evident upon treatment with 10 μM isoproterenol (Fig. 5*F*, top panel, lane 3). Control immunoblots confirmed equivalent levels of total USP4 and SERCA2 in all samples (Fig. 5*F*, upper-mid and lower mid panels). The influence of AKAP18 on USP4 phosphorylation was established by RNA knockdown (Fig. 5*F*, bottom panel). Gene silencing of AKAP18 markedly reduced the pSer^829^ signal in cells treated with isoproterenol (10 μM) as compared to scrambled shRNA controls (Fig. 5*F*, top and bottom panels, lanes 3 and 4). Collectively these findings show that AKAP18 anchored PKA phosphorylates serine 829 on USP4. This residue is adjacent to the active site.

### *In situ* detection of phospho-Ser^829^ on USP4

Cell-based evaluation of USP4 phosphorylation was conducted in two ways. Immunofluorescent imaging monitored the impact of adrenergic stimulation on pSer^829^ modification of USP4. Isolated mouse adult ventricular myocytes were pretreated with the adrenergic agonist Isoproterenol, or the β-blocker drug propranolol (Fig. 5, *G* and *H*). Cells were fixed with paraformaldehyde and stained with antibodies against SERCA2 (cyan), total USP4 (green), and pSer^829^ USP4 (magenta). The pSer^829^ USP4 signal was not evident upon pretreatment of cardiomyocytes with propranolol (Fig. 5*G* gray scale bottom inset). In contrast, the pSer^829^ USP4 signal was detected upon adrenergic stimulation with isoproterenol (Fig. 5*H* gray scale bottom inset). SERCA2 and total USP4 staining displayed similar patterns and signal intensities under both treatment conditions (Fig. 5, *G* and *H*, gray scale insets). Composite images show enrichment of the pSer^829^ USP4 signal with SERCA2 at the periphery and in the Z bands upon treatment with isoproterenol (Fig. 5*H*). These imaging experiments suggest that mobilization of the cAMP signaling pathway enhances phosphorylation of Ser 829 of USP4.

The heart undergoes extensive cellular remodeling and altered kinase signaling after myocardial infraction (MI) (80). Therefore, anti-pSer^829^ antisera were used to screen pathological sections of normal and diseased human left ventricular heart tissue. In normal heart tissue the pSer^829^ USP4 signal was low and uniformly distributed throughout cardiomyocytes (Fig. 5*I*, top and bottom panels). In contrast, the pSer^829^ USP4 signal was robust and concentrated at the Z bands in tissue sections collected 5 to 7 days post MI (Fig. 5*J*, all panels). Increased detection of pSer^829^ USP4 in post MI tissue sections may represent a restorative mechanism to maximize deubiquitinase activity in the vicinity of Z bands.

### AKAP18 coordinates PKA modulation of SERCA2

**Figure 6:**
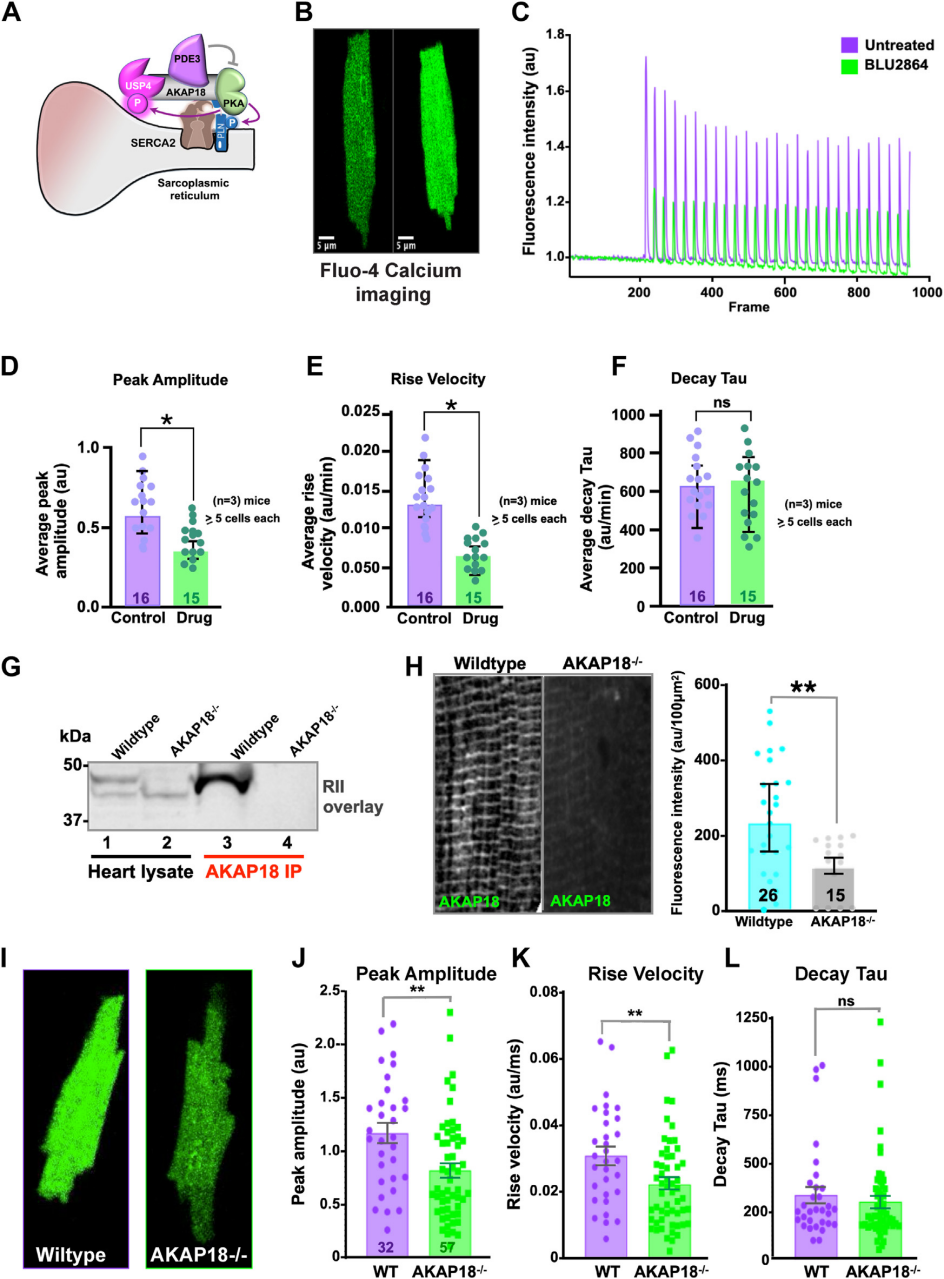
**AKAP18 coordinates aspects of PKA modulation of SERCA2.***A*, diagram of an AKAP18 signaling island at the sarcoplasmic reticulum. PKA (*green*), SERCA2 (*brown*), phospholamban (*teal*), and USP4 (*magenta*) are indicated. Phosphodiesterase 3 (PDE3, *purple*) terminates cAMP signals. *Arrows* indicate sites of anchored PKA phosphorylation. *B*, mouse adult cardiomyocytes loaded with Fluo-4 calcium indicator dye were paced at different frequencies (1–4 Hz) with field stimulation. Representative images of (*left*) low and (*right*) high calcium transients in pulsing cells. Scale bars (5 μm) are indicated. *C*, time course (sec) of calcium transient florescence intensities (494/506 nm) for control (*purple*) and BLU2864-treated (*green*) cardiomyocytes. *D*–*F*, amalgamated data (>5 cells per animal n = 3) showing changes in control (*green*) and BLU2864 treated cardiomyocytes. Graphs showing changes in (*D*) peak amplitude (calcium transient relative to resting value); (*E*) rise velocity (rate of the increase of calcium in the cytosol) and (*F*) decay tau (kinetics of calcium clearance) are presented. Numbers of individual cells shown measured below each column. *G* and *H*, characterization of AKAP18^−/−^ mice. *G*, RII overlay is a modified Western blot procedure used for the detection of AKAPs. Heart lysates (lanes 1 and 2) and AKAP18 immune complexes (lanes 3 & 4) obtained from WT and AKAP18^−/−^ mice (indicated above each lane) were probed for AKAPs. Molecular weight markers are indicated. *H*, immunofluorescence detection of AKAP18 in cardiac tissue sections. Representative images from (*left*) WT and (*right*) AKAP18^−/−^ mice are included. Quantification of fluorescent signal by Fiji/ImageJ (au/100 μm^2^) for WT (*cyan*) and AKAP18^−/−^ tissue (*gray*). Number of cells used in each analysis are indicated. *I*, representative images maximal calcium transients of WT (*left*) and AKAP18^−/−^ (*right*) in cells. *J*–*L*, amalgamated data (numbers of cells shown below each column; n = 5 animals) showing phenotypic differences between WT (*purple*) and AKAP18^−/−^ (*green*) cardiomyocytes. Graphs showing changes in (*J*) peak amplitude (calcium transient relative to resting value); (*K*) rise velocity (rate of the increase of calcium in the cytosol) and (*L*) decay tau (kinetics of calcium clearance) are presented. All statistical measurements are presented as means ± SEM. AKAP18, A-kinase anchoring protein 18; PKA, protein kinase A; SERCA2a, sarcoplasmic reticulum through the Ca^2+^ATPase 2a; USP, ubiquitin-specific proteinase.

Phospholamban and sarcolipin are regulators of SERCA2 during myocardial contraction (Fig. 6*A*). Phospholamban is a principal mediator of β-adrenergic responses (55). AKAP18γ coordinates PKA phosphorylation of phospholamban during calcium reuptake ((67, 81) Fig 6*A*). This proceeds through PKA phosphorylation of Ser 16 on phospholamban to increase SERCA2 pump activity ((82) Fig 6*A*). Therefore, we reasoned that inhibition of the kinase or ablation of the *AKAP7 (AKAP18)* gene would have functional consequences on calcium dynamics. Freshly isolated left ventricular cardiomyocytes were loaded with the fluorescent calcium indicator, Fluo-4 AM, and changes in calcium dynamics were imaged in an Airyscan confocal microscope (Fig. 6*B*). Cardiomyocytes were paced at different frequencies (1–4 Hz) with field stimulation. Calcium transients were normalized by baseline fluorescence (F1/F0) and analyzed using Clampfit software (83, 84). Vigorous calcium transients were observed in control myocytes (Fig. 6*C*; purple; 5–8 cells each from three animals). Importantly, peak amplitude and time to the peak were attenuated upon application of BLU2864 (10 nM), a selective PKA inhibitor (Fig. 6, *D* and *E*, green; 5–8 cells each from three animals). Conversely, the decay tau was similar in control and drug-treated cardiomyocytes (Fig. 6*F*), suggesting no effects on calcium reuptake. Thus, pharmacologically blocking PKA action reduces calcium flux through SERCA2. Next, calcium transients were measured in cardiomyocytes from conditional AKAP18^−/−^ mice. Deletion of all AKAP18 isoforms in knockout cardiomyocytes was confirmed by RII overlay in heart lysates and AKAP18 immune complexes (Fig. 6*G*). Immunofluorescent staining independently established loss of the anchoring protein in AKAP18^−/−^ cardiomyocytes as compared to WT (Fig. 6*H*). Calcium dynamics from AKAP18^−/−^ cardiomyocytes showed reduced peak amplitudes and time to the peak compared to WT animals (Fig. 6, *I*–*L*). The decay tau was similar in both genotypes suggesting that AKAP18 mediated effects were in the early phases of SERCA2 action (Fig. 6*L*).

## Discussion

In this report, we show that the ubiquitin-specific protease, USP4, is anchored with PKA in the vicinity of the sarcolemma Ca^2+^ ATPase (SERCA 2). Coclustering of these enzymes *via* interaction with AKAP18 permits coincident regulation of protein ubiquitination and protein phosphorylation in the heart. The signal termination enzyme USP4 not only protects against ubiquitin mediated protein degradation but can also expose interactive surfaces that are normally occluded by mono or polyubiquitin chains. USP4 only interacts with the γ and δ isoforms of AKAP18, and chemical cross-linking implicates a 2′phosphoesterase (CD) domain as the principal interactive unit on the anchoring protein. Isolation of only a few cross-link sites at the periphery of the CD domain is compatible with the putative model for AKAP18-USP4 docking put forward in Figure 4*G*. Additional structural experiments will be necessary to validate this configuration. It will also be interesting to establish if the related deubiquitinates USP7 and USP10 detected in our AKAP18γ proteomic screens dock in a similar manner (Fig. 4*D* & (16)). The 2′phosphoesterase fold is an ancient domain which is present in enzymes that hydrolyze cyclic nucleotides; yet, there is no evidence that AKAP18 can metabolize cAMP (72, 73, 85). Rather, it is more likely that the CD domain of AKAP18 acts as an AMP sensor. This nucleotide is released upon metabolism of cAMP by another AKAP18-binding partner, phosphodiesterase 3A (43, 72). Alternatively, incorporation of AMP may stabilize the CD domain to favor USP4 binding, or act as an allosteric modulator of deubiquitinase activity. Irrespective of which mechanism predominates, sarcoplasmic AKAP18 anchored PKA and PDE3 acutely control cAMP responsive phosphorylation of phospholamban and SERCA2, whereas sequestered USP4 terminates ubiquitin signaling over a longer time frame (14, 17, 82).

Several lines of evidence indicate that USP4 activity plays a cardioprotective role (86). USP4 depletion exacerbates cardiac dysfunction in mice subjected to pressure overload (87). Pathological cardiac hypertrophy is reduced in transgenic animals that overexpress this deubiquitinase (88). One USP4 target may be SERCA2 as the ubiquitin–proteasome system degrades proteins that mediate calcium homeostasis (60). Paradoxically, conjugation of the SUMO augments SERCA2 protein stability (89). Hence, USP4 may be anchored at sarcomeres to remove ubiquitin from already SUMOylated calcium exchangers. Since deubiquitination counteracts the action of E3 ligases it is inevitable that proteostatic control of SERCA2 must involve this later class of enzyme. The UBR family of E3 ligases performs various functions in the cardiovascular system, of which UBR3 and UBR6 influence the amplitude of sarcoplasmic Ca2+ release (60). The identification of UBE2O, UBA1, and RNF213, in the proximity proteomics screen reported in Figure 2*D* suggests that these E3 ligases may be operational in the vicinity of AKAP18. Likewise, NEDD4 family E3 ligases modify a range of clients, including SERCA2a (90). Interestingly these E3 ligase classes also target Ca_v_1.2 channels to dampen adrenergic stimulation of excitation–contraction coupling (91). Given that SERCA2a resides within 50 to 100 nm of the calcium channel it is reasonable to postulate that AKAP18 associated pools of USP4 are equally well positioned to remove ubiquitin from both calcium effectors (92).

Phosphoproteomic studies indicate that USPs are phosphorylated (93). This can have varied effects. Phosphorylation within the WW domain augments the catalytic efficiency of USP1, tyrosine or serine phosphorylation biases substrate recognition of USP7, and glycogen synthase kinase 3 mediated phosphorylation of USP27 stabilizes this enzyme (94, 95). Molecular modeling data presented in Figure 5 suggest that PKA phosphorylation of USP4 occurs at serine 829 in a cleft proximal the active site. The added bulk or negative charge provided by pSer^829^ may alter the catalytic core to improve recognition of ubiquitinated clients. Data with phosphopeptide antibodies presented in Figure 5*G* show that adrenergic stimulation enhances pSer^829^ labeling in cardiomyocytes. This argues that AKAP18-anchored PKA has preferred access to USP4 and that the signaling complex must be located close to sites of cAMP generation (23). Two of our findings support this claim. First, data presented in Figure 5 show that gene silencing of AKAP18 reduces cAMP responsive phosphorylation of USP4. Second, adrenergic stimulation of adult cardiomyocytes enhanced *in situ* detection of pSer^829^ at Z bands and periphery of these cells. This argues that AKAP18-USP4 subcomplexes reside close to beta 2 adrenergic receptors which populate the t tubules located just above the Z bands (4, 82). Thus, USP4 proximity to PKA seems to be a determinant in the cAMP dependent modulation of this sarcoplasmic deubiquitinase. Other cardiac AKAP signaling islands may also participate in this process. AKAP79/150 sequesters β2 adrenergic receptors with PKA and the adenylyl cyclase AC5 and AC6 isoforms. This autonomous cAMP signaling unit responds rapidly to pulses of adrenergic stimuli (96, 97). AKAP79/150 also associates with the cytoplasmic tail of Ca_v_1.2 channels, whereas synemin and myospryn sequester PKA at the Z-lines within range of the calcium channel (98, 99). Hence, the concerted action of distinct AKAP signaling islands may provide precise spatial control of PKA at the sarcomere. However, a unique facet of the AKAP18 signaling complex is that temporal control is conferred by phosphodiesterase 3A, a binding partner that rapidly degrades cAMP (43, 81). Accordingly, peptidomimetics targeting the AKAP18δ have been developed as therapeutic agents to control calcium reabsorption at the sarcoplasmic reticulum in diseased hearts (81). Processive engagement of distinct AKAP signaling islands during excitation–contraction coupling may be an underappreciated feature of the adrenal response.

Suppression of USPs is emblematic of maladaptive cardiac hypertrophy and aspects of congestive heart failure (88, 100, 101). Conversely, mechanisms that acutely augment USP4 activity such as phosphorylation of Ser 829 may help to restore damaged hearts. The concomitant deubiquitination of sarcoplasmic proteins not only safeguards against proteasomal processing of SERCA2 but will also preserve any of the cardioprotective SUMOylated forms of the calcium exchanger (57, 89). Alternatively, increased detection of phospho-USP4 in human post MI tissue (Fig. 5*J*) may be a consequence of elevated PKA activity that occurs as a response to arterial blockage (102). It is also worth noting that anchored PKA and anchored USP4 effect SERCA2 with different timescales. Live cell imaging data in Figure 6, *B*–*F* show that application of the selective PKA inhibitor compound BLU2864 instantly attenuates calcium transients in adult cardiomyocytes in a millisecond time frame (103). Conversely, prolonged treatment with the USP4/USP5 antagonist vialinin A has a minimal effect in the same experimental system. Thus, regulation by USP4 is most likely a more protracted process, requiring protein translation, unfolding, and degradation.

Similar effects were observed when calcium transients were measured in adult cardiomyocytes isolated from AKAP18^−/−^ mice. The reductions in peak amplitude and rise velocities of calcium transients presented in Figure 6, *J* and *K* represent the first phenotypic effects reported in AKAP18^−/−^ mice. Earlier electrophysiological studies concluded that Ca_v_ 1.2 channels respond normally to adrenergic stimulation in AKAP18^−/−^ cardiomyocytes (104). There are several explanations for these differences. Monitoring transient rises in cytosolic Ca^2+^ concentrations occur in part because of calcium release and reuptake through SERCA2 (46). This cAMP responsive process is under the control of AKAP18γ and AKAP18δ at the sarcomere (43, 81, 105, 106). In contrast, adrenergic modulation of Ca_v_ 1.2 currents occurs at the plasma membrane. Several AKAPs have been implicated in the control of this latter signaling event. Initially AKAP79/150 complexes were shown to coordinate modulation L type calcium currents by physically associating with the cytoplasmic tail of the ion channel (40, 107, 108). Subsequent studies have implicated ⍺ and β isoforms of AKAP18 and Cypher/Zasp as channel-associated anchoring proteins that participate in this process (62, 71, 109, 110). However, local PKA modulation of Ca_v_1.2 by the Rem and Rad small GTPases is now accepted as the principal means of unleashing the adrenergic response in cardiomyocytes (39, 111). Hence, any combination of these redundant channel activation pathways could compensate for ablation of the AKAP18 and AKAP79/150 genes in mice (30, 79, 104). Nevertheless, our study concludes that AKAP18 plays a unique role in organizing signaling enzymes that engage the protein phosphorylation and ubiquitin pathways to efficiently control of adrenergic control of cardiac contractility.

## Experimental procedures

### Isolation of rat neonatal cardiomyocytes

Neonatal cardiomyocytes (CMs) were isolated enzymatically from 2- to 3-day-old Sprague-Dawley rat heart and enriched *via* percoll gradient centrifugation (37). Isolated CMs were seeded onto cell culture plates and cultured in medium containing Dulbecco's modified Eagle's medium-F10 with 10% horse serum, 5% fetal bovine serum, 1% penicillin/streptomycin at 37 °C in a humidified atmosphere containing 5% CO_2_. Neonatal CM purity was evaluated by immunocytochemistry with alpha-actinin (staining for Z disk in sarcomeres) and shown to be over 90%.

### MS sample preparation and analysis for proximity proteomics

CMs were infected with adenoviruses 48 h post isolation (Multiplicity of infection: GFP-mT = 8, AKAP18γ-mT = 80), switched to serum-free media, and on day 6 post isolation were incubated with 50 μM biotin (Sigma-Aldrich, B4501) for 3 h. Cells were processed for MS analysis by the NBCC Proteomics Core at the Lunenfeld-Tanenbaum Research Institute as described (112). Frozen cell pellets were lysed using radioimmunoprecipitation assay lysis buffer (50 mM Tris–HCl (pH 7.5), 150 mM NaCl, 0.1% (w/v) SDS, 1% NP-40, 1 mM MgCl_2_, 1 mM EDTA, 0.5% (w/v) sodium deoxycholate, and 1x Sigma protease inhibitors), sonicated, and treated with 10 μg RNase at 4 °C for 30 min. Lysates were applied to 30 μl of prewashed streptavidin beads (Cytiva 17–5113–01) for 3 h, washed, and digested with 1 μg of trypsin in 50 μl of 50 mM ammonium bicarbonate, overnight at 37 °C. Additional trypsin (0.5 μg) was added and incubated at 37 °C for 4 h. Formic acid was added to a final concentration of 5%, peptides were desiccated and stored at −40 °C for MS analysis. For data-dependent acquisition by liquid chromatography with tandem mass spectrometry, affinity purified and digested peptides were analyzed using a nano-HPLC coupled to MS, as described for data set.

MS data were analyzed using ProHits laboratory information management system platform. The data were then searched using Mascot (V2.3.02) and Comet (V2016.01 rev.2). The spectra were searched with the rat sequences in the RefSeq database (version 97) acquired from NCBI, supplemented with “common contaminants” from the Max Planck Institute (http://maxquant.org/contaminants.zip) and the Global Proteome Machine (GPM; ftp://ftp.thegpm.org/fasta/cRAP/crap.fasta), forward and reverse sequences (labeled “gi|9999” or “DECOY”), sequence tags (GFP, BirA, GST26, and mCherry), streptavidin, and LYSC_PSEAE for a total of 134,277 entries. Results from each search engine were analyzed through TPP (the Trans-Proteomic Pipeline, v.4.7 POLAR VORTEX rev 1) *via* the iProphet pipeline (113). Proteins were filtered based on iProphet probability ≥ 0.95 and unique peptides ≥ 2. The SAINT analysis tool was used to identify high-confidence protein interactors *versus* control samples (114) SAINTexpress: improvements and additional features in SAINT software. SAINTexpress (version 3.6.1) was used to calculate the probability that identified proteins were enriched above background contaminants. Bait protein (*e.g.*, AKAP18) was profiled using independent biological triplicates and analyzed alongside 14 independent negative controls (biotin alone with no infection (3 replicates) and GFP-mT (11 replicates)). For analysis with SAINT, samples were processed through the iProphet pipeline and filtered by probability of >0.95. Minimum of two unique peptides were required for inclusion.

### Deubiquitinase assay

Ub-AMC (ubiquitin-AMC, UbiQ-001) deubiquitinase assay was performed following manufacturer instructions using the method reported by Dang et al (115). Ub-AMC is a quenched fluorogenic substrate for DUB enzymes. It is based on ubiquitin that is functionalized with a C-terminal 7-amido-4-methylcoumarin (AMC). Cleavage of the amide bond between the C-terminal Gly and AMC releases the fluorescent dye (excitation 380 nm, emission 460 nm).

### Data and statistical analyses

Excel (Microsoft) and Prism (GraphPad) were used to analyze data. ImageJ was used to process images. *t* Test and one-way ANOVA were used to test for statistical significance. *p*-values <0.05 were deemed statistically significant. The number of cells or samples used for each experiment is detailed in each figure. In all experiments at least three biological replicates were performed. All measurements presented as means ± SEM.

### Isolation of mouse adult ventricular myocytes

We followed the previously published method of Santana, Moreno et al., with slight modifications (116). Mouse hearts were rinsed in 50 ml of 25 mM Hepes, 130 mM NaCl, 5 mM KCl, 22 mM glucose, 3 mM pyruvic acid, 0.5 mM MgCl_2_, and NaH2PO_4_ buffer pH 7.4. The ventricles are removed and dissected to make a single cell cardiomyocyte mixture. Supernatant is collected into a 15 ml conical tube and allowed to settle at room temperature for 10 to 20 min. Cells were used for proximity ligation, immunofluorescent staining, and calcium imaging analyses.

### Proximity ligation

PLA visualizes the subcellular location of protein-protein interactions at endogenous levels. This is achieved using antibody probes that recognize target proteins within 40 nm of each other (117). The probes are ligated together, hybridizing to form a circular DNA. Fluorescent- or horseradish peroxidase (HRP)-labeled oligonucleotides. PLA was performed on isolated adult cardiomyocytes using our previously published method (25). Bethyl Laboratories, 1:1000 dilution Goat anti-AKAP18 (lot no 3265) and Bio-Techne anti Human/Mouse RII⍺ (Catalog #: MAB8000) were used for RII/AKAP18 pairs. 1:500 dilution Rabbit polyclonal USP4 antisera (Novus Biologicals, NBP1-86876) was paired with 1:500 dilution Goat anti-AKAP18 to detect anchoring protein/deubiquitinase puncta.

### Immunoprecipitation, immunoblotting, and autoradiography

HCM (PromoCell) or HEK293T (GE Healthcare) cells were transfected with 3 μg DNA per 10 cm dish using TransIT-LT1 (Mirus). After 48 h cells were harvested in lysis buffer (25 mM Hepes, pH 7.4, 150 mM NaCl, 1 mM EDTA, 1 mM EGTA, 20 mM NaF, 2% glycerol, and 1% Triton X-100) containing protease inhibitors. AKAP18 complexes were immunoprecipitated with rabbit anti-GFP IgG (Invitrogen) and protein A agarose for 2 h at 4 °C. Beads were washed 4 × 1 ml in lysis buffer. Proteins were separated on 4 to 12% gradient gels (Invitrogen) and transferred to nitrocellulose membranes. Immunoblot analyses followed our previously published protocol (16). For experiments in Figure 1*F*, SERCA2 was immunoprecipitated from cleared lysates using Novus Biologicals Mouse Monoclonal NB300 to 581, (Clone 2A7-A1). Immunoblot detection of USP4 was with rabbit polyclonal antisera 1:500 dilution (Novus Biologicals, NBP1-86876) and PKAc with 1:1000 dilution of mAb (BD Biosciences clone 5B). For experiments in Figure 4*B*, AKAP18 isoforms were detected with 1:1000 dilution rabbit anti-GFP IgG (Invitrogen) and 1:1000 dilution monoclonal antibodies against V5 (TCM5; Thermo Fisher Scientific). For experiments in Figure 5, *A* and *E*, immunoblot detection of USP4 was with rabbit polyclonal antisera 1:500 dilution (Novus Biologicals, NBP1-86876). AKAP18 was detected with 1:1000 dilution of a custom antibody generated by (Bethyl Laboratories) (16). RII was detected with 1:500 dilution of a custom antibody (118). In Figure 5*A* Human cardiac myocytes HCM (PromoCell) lysates were incubated with 1 nM ^32^P ATP. Fractionation of ^32^P-labeled proteins was detected by autoradiography upon immunoprecipitation of USP4 with rabbit polyclonal antibodies (Novus Biologicals lot; NBP1-86876). Membranes in all experiments were washed extensively in Tris buffered saline 1% tween 20 (TBST), incubated with HRP-labeled secondary antibodies (Jackson ImmunoResearch), washed as before, and developed using ECL (Thermo Fisher Scientific) on an Alpha Innotech Multi Image III with FluoroChem Q software, or with an iBright FL1000 imager (Thermo Fisher Scientific). For reprobing, membranes were stripped with 1X ReBlot Plus Strong (Millipore) for 15 min and then reblocked in Blotto before incubation with primary antibodies again. GST pull-downs were performed as described previously (119).

### Immunostaining

Immunofluorescence was performed following our previously published protocols with slight modifications (120). CMs were seeded at a density of ∼50,000 cells per well on a 24-well plate containing a 12 mm #1.5 cover glass and incubated overnight. Specimens were fixed for 10 min in a solution containing 3.2% paraformaldehyde and 0.1% glutaraldehyde in polyelectrolyte multilayer (for microtubules) or PBS, followed by brief washing in PBS and reduction in an aqueous solution of 10 mM sodium borohydride for 5 min. Samples were washed three times with PBS and then incubated with blocking/permeabilization buffer (PBS with 5% bovine serum albumin (BSA) and 0.5% Triton X-100) for 30 min. Samples were incubated with primary antibodies in blocking/permeabilization buffer for 45 min, washed three times with PBS, and incubated for 45 min with 1:1000 dilution secondary antibodies.

### Pull-down experiments

HEK293 cells were transfected with 2 μg of plasmid DNA (pcDNA3, USP4-myc, or USP7-myc) per 60 mm dish using Lipomaster 2000 reagent (Vazyme) according to the manufacturer’s protocol. Transfected cells were incubated for 48 h, followed by infection with adenoviral AKAP18γ-mT (Multiplicity of infection = 1.5). After 24 h, cells were harvested and lysed in lysis buffer containing 20 mM Hepes (pH 8.0), 1 mM EDTA, 1 mM MgCl_2_, 1 mM DTT, 150 mM NaCl, 0.5% C_12_E_10_, and protease inhibitors. Lysates were clarified by centrifugation, and the supernatants were incubated with MYC primary antibody (1:300) at 4 °C for 2 h, followed by incubation with protein A/G agarose beads (Santa Cruz Biotechnology) for 1 h. The resin was washed three times with wash buffer (20 mM Hepes, pH 7.4, 1 mM EDTA, 1 mM MgCl_2_, 110 mM NaCl, 0.05% C_12_E_10_, and protease inhibitors). Bound proteins were eluted by resuspending the beads in SDS-PAGE sample buffer and heating at 60 °C for 10 min. Proteins were separated by SDS-PAGE using NuPAGE 4 to 12% polyacrylamide gels and transferred onto a polyvinylidene fluoride membrane (Thermo Fisher Scientific). Membranes were blocked in 5% BSA in Tris-buffered saline with 0.1% Tween-20 (T-TBS) and probed with anti-FLAG, anti-MYC, and anti-β-actin antibodies. Protein signals were detected using SuperSignal chemiluminescent reagent (Thermo Fisher Scientific) and visualized with an Odyssey imaging system (LI-COR Biosciences).

### Chemical cross-linking

PIR cross-linking with MS permits structural biology measurements in biological samples. Experiments were conducted following the protocol of Chavez et al (75). Cross-linking of purified USP4 to the central domain of AKAP18 (both over a range of concentrations 01.-1 mM) was conducted in 25 mM sodium phosphate buffer pH 7.4 with 1 mM PIR cross-linker. Digestion with trypsin cleaves at Arg and Lys residues. Lys residues reacted with the cross-linker resulting in missed tryptic cleavage sites. Cross-linked peptide pairs were fractionated on reverse phase HPLC using a C8 column and subjected to MS analyses on a Q-Exactive Plus from Thermo Fisher Scientific LC–MS instrument. Identification cross-linked peptide sequences used xQuest algorithm (76).

### Molecular modeling

Crystal structures of AKAP18 and USP4 were retrieved from protein data base (PDB: 2VFY for AKAP18 and 2Y6E for USP4). Structures were arranged the PyMOL 3.0 (Schrödinger) (121)). For Figure 4*G* residues were highlighted to correspond with cross-linking data. For Figure 5*C* a space filling model of USP4 active site was generated.

### Generation of phospho-peptide USP4 antibodies

Rabbit anti phospho-USP4 antibodies were generated under contract from New England Peptide (Lot no 2282–20/21). A synthetic Ac-CHLKRSYNRYWR-amide peptide was conjugated to KLH and injected into animals monthly thrice. Antibodies were affinity purified using the original antigen conjugated to resin.

### Peptide spot array synthesis

SPOT arrays were generated as described previously (122). FMOC amino acids were purchased from AnaSpec and cellulose membranes for SPOT synthesis were purchased from Intavis. Peptides were synthesized onto a membrane using the Intavis MultiPep solid-phase peptide synthesizer following the manufacturer’s standard protocols.

### Gene silencing of AKAP18

RNAi knockdown of AKAP18 isoforms was performed with AKAP7 shRNA lentiviral particles (Santa Cruz, sc-95270). Cells were infected with virus and incubated for 48 h at 37 °C. Control cells were infected with scrambled shRNA particles (sc-108080). Depletion of AKAP18 was confirmed by immunoblot as described above using goat antibodies against the anchoring protein (Bethyl Laboratories, 3265).

### RII overlay

His-tagged mouse RII⍺ was expressed in BL21(DE3) pLysS *Escherichia coli* and purified by Ni + -affinity and size-exclusion chromatography as described. Purified RII was then biotinylated using EZ-Link-Sulfo-NHS-biotin (Thermo Fisher Scientific) according to the product protocol. Biotinylated RII was incubated with peptide array membranes overnight at 4 °C in Tris buffered saline 1% tween 20 containing 5% nonfat dry milk and 1% BSA. After extensive washing, the membrane was incubated with neutravidin-HRP (Thermo Fisher Scientific), washed again and imaged by ECL (Super-signal Pico, Thermo Pierce) on an Alpha Innotech Multi Imager III. Quantification using densitometry of RII overlay signal was performed using Fiji/ImageJ and normalized to corresponding WT peptide signal.

### Deparaffinization of embedded heart tissue

In preparation for immunohistochemical staining, paraffin-embedded (FFPE) tissue sections were treated to remove the paraffin and unmask epitopes following the method of Shi et al., with slight modifications (123). Coverslips containing paraffin embedded pathological samples were incubated for 10 min in xylene solution at room temperature. After draining, samples were washed two times for 10 min at room temp in 100% ethanol. This was followed by washes in 95% and 80% ethanol and proceeded by two further washes (5 min) in distilled water. Immunofluorescent staining of tissue sections was as described above.

### Generation of AKAP18^−/−^ mice

Deletion of the AKAP7 gene was achieved as originally described by Jones et al, (104). Heterozygous AKAP18 conditional KO mice were rederived from the MMRRC facility at UC Davis (B6.Cg-Akpa7tm1.2Gsm/Mmucd, MMRRC:036972-UCD). Mutant animals were crossed to EIIa-Cre mice on a C57Bl6/J background, from Jackson Labs, to obtain AKAP18^+/−^ mice. These mice were subsequently intercrossed to obtain the AKAP18^−/−^ mice.

### Animals

All experimental procedures and protocols were approved by the Animal Care and Use Committee of the University of Washington and conformed to the National Institute of Health “Guide for the Care and Use of Laboratory Animals”.

### Calcium dynamics

CMs were loaded with 5 μM of the acetoxymethyl ester form of the calcium indicator Fluo-4 (Fluo4-AM, Invitrogen), mixed with pluronic acid, for 30 min at 37 °C in a CO_2_ incubator. After incubation, the cells were rinsed in Tyrode's solution to remove any excess dye before imaging. APs were evoked *via* field stimulation at a frequency of 1 to 4 Hz using an IonOptix MyoPacer (IonOptix Corp), which delivered square voltage pulses at an amplitude of 20 V. AP-evoked calcium signals were imaged using an AiryScan system (Zeiss LSM 880) equipped with a Plan-Apochromat 63X/1.40 NA oil immersion objective. The system was controlled by ZEN black v2.3 software in confocal mode. Fluo-4 was excited with a 488 nm laser, and the emitted light was collected from 495 to 600 nm. Time series were captured at intervals of 100 ms, while bath perfusion during imaging was regulated through a gravity-driven system at a flow rate of 2 ml/min. Images were analyzed using ImageJ (NIH) to extract data on the time course of changes in fluorescence intensity. The data was then exported and further analyzed using Clampfit (Molecular Devices) to extract dynamic parameters, including peak amplitude, time to peak, and decay phase.

## Data availability

Data have been deposited as a complete submission to the MassIVE repository (https://massive.ucsd.edu/ProteoSAFe/static/massive.jsp) and assigned the accession number MSV000096656. The dataset is accessible at ftp://massive-ftp.ucsd.edu/v07/MSV000096656/. The ProteomeXchange accession is PXD058849.

## Supporting information

This article contains supporting information.

## Conflict of interest

The authors declare that they have no conflicts of interest with the contents of this article.
