# Supplementary material for: Long AKAP18 isoforms anchor ubiquitin specific proteinases and coordinate calcium reuptake at the sarcoplasmic reticulum
Source: J Biol Chem. 2025 May 29;301(7):110317. doi: 10.1016/j.jbc.2025.110317 (PMC12221368; doi:10.1016/j.jbc.2025.110317)

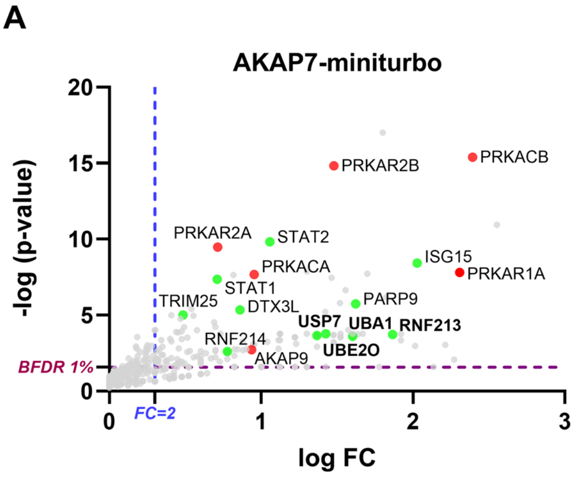

Figure S1. A, Volcano plot of proximity proteomics for AKAP7-miniturbo compared to GFP-miniturbo and non-infected controls. All samples were prepared in triplicate from neonatal CMs. The $\log_{10}$(fold change) was plotted versus $-\log_{10}$(p-value), with the Bayesian false-discovery rate (BFDR, 1%) and fold change (FC, 2) indicated by dashed lines. The p-value was calculated using paired student t-test. AKAP7 near neighbor proteins corresponding to protein kinase A binding (red) and ubiquitination (green) are shown. **B,** AKAP7-miniturbo gene ontology enrichment for biological process (top right), molecular function (lower left) and cellular component (lower right) are shown. Enrichment analysis was performed using STRING (version 12.0) on near neighbor proteins with a SAINT cut-off score of SS≥0.7, BFDR≤3%, and FC>2.

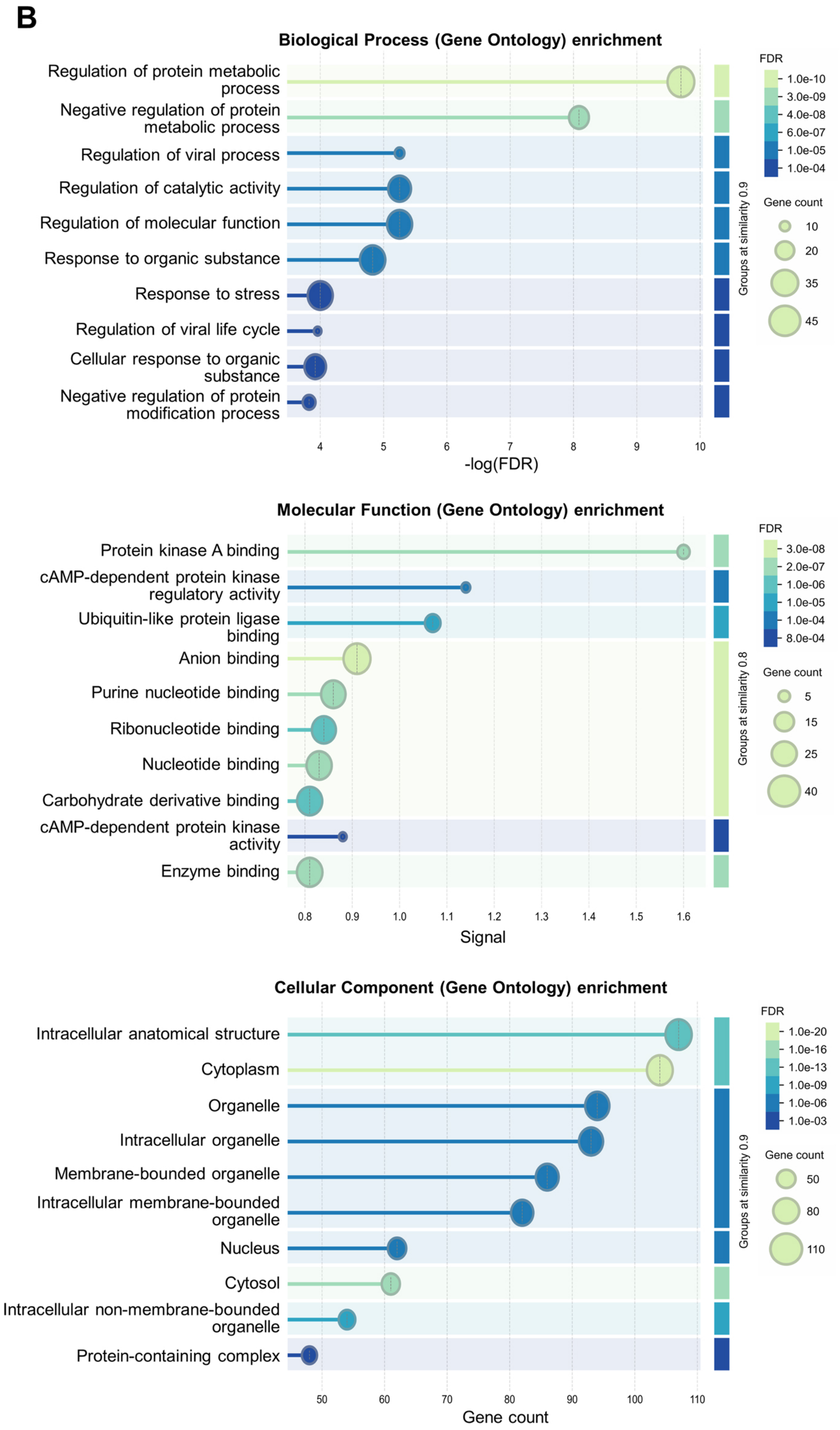

Supplement: Figure S1 [file mmc1.pdf]
